# Fluoride in Dental Caries Prevention and Treatment: Mechanisms, Clinical Evidence, and Public Health Perspectives

**DOI:** 10.3390/healthcare13172246

**Published:** 2025-09-08

**Authors:** Chin-Hsuan Yeh, Yung-Li Wang, Thi Thuy Tien Vo, Yi-Ching Lee, I-Ta Lee

**Affiliations:** 1 Division of Oral and Maxillofacial Surgery, Department of Dentistry, Taipei Medical University Hospital, Taipei 110301, Taiwan; 163017@h.tmu.edu.tw; 2 School of Dentistry, College of Oral Medicine, Taipei Medical University, Taipei 110301, Taiwan; cetuswang@tmu.edu.tw; 3 Faculty of Dentistry, Nguyen Tat Thanh University, Ho Chi Minh City 700000, Vietnam; vtttien@ntt.edu.vn; 4 Center for General Education, Chang Gung University of Science and Technology, Chiayi 61363, Taiwan

**Keywords:** fluoride interventions, dental caries prevention, remineralization and demineralization, public health and cultural perspectives, clinical efficacy and safety

## Abstract

**Background:** Dental caries remains one of the most prevalent chronic diseases worldwide. Fluoride has long been recognized as a cornerstone of caries prevention through enamel remineralization, inhibition of demineralization, and antibacterial activity. However, controversies persist regarding systemic exposure, potential health risks, and ethical debates over community water fluoridation. Previous reviews often focused on isolated interventions, whereas a critical synthesis of mechanisms, clinical efficacy, safety, and public health perspectives is still lacking. **Methods:** This narrative review synthesized peer-reviewed publications from 2000 to 2025 retrieved from PubMed, Scopus, Web of Science, and leading dental journals. Emphasis was placed on randomized controlled trials, systematic reviews, meta-analyses, and major policy documents. Evidence was thematically appraised across mechanisms of action, clinical applications, comparative efficacy, safety, and sociocultural considerations. **Results:** Fluoride consistently shows preventive and therapeutic benefits across multiple delivery forms, including toothpaste, varnishes, mouthrinses, supplements, and silver diamine fluoride, with particular advantages for high-risk groups such as children, orthodontic patients, and older adults. Nonetheless, study heterogeneity, variations in protocols, and concerns regarding fluorosis and possible neurodevelopmental effects highlight persistent uncertainties. Comparative analyses reveal trade-offs between efficacy and acceptance, for example, the high caries-arrest rate of silver diamine fluoride compared with its esthetic drawback. Emerging alternatives such as nano-hydroxyapatite, fluoride-containing bioactive glass, and probiotic-based approaches are promising but currently supported by limited clinical data. **Conclusions:** Fluoride remains central to caries prevention, yet its optimal use requires balancing benefits against risks, addressing cultural and socioeconomic barriers, and tailoring strategies to individual and community contexts. This narrative synthesis underscores the need for well-designed multicenter randomized controlled trials, longitudinal studies to refine safe exposure thresholds, evaluations of novel biomaterials and delivery systems, and the incorporation of patient-reported outcomes to guide future evidence-based policies and clinical practices.

## 1. Mechanisms of Action of Fluoride

The primary mechanism by which fluoride prevents dental caries lies in its capacity to promote enamel remineralization and inhibit demineralization. Tooth enamel is mainly composed of hydroxyapatite (Ca_10_(PO_4_)_6_(OH)_2_), a crystalline calcium phosphate that is relatively susceptible to acid dissolution during pH fluctuations caused by bacterial metabolism of dietary carbohydrates [1]. When fluoride ions are present, they can partially replace hydroxyl groups in hydroxyapatite, leading to the formation of fluorohydroxyapatite, an intermediate phase that exhibits greater stability and reduced solubility compared with pure hydroxyapatite. With more extensive substitution, fluorapatite (Ca_10_(PO_4_)_6_F_2_) is formed, which has the lowest solubility and the highest resistance to acid attack. This transformation effectively reduces the critical pH for demineralization from approximately 5.5 to about 4.5, making enamel more resistant to caries development [2,3]. Moreover, even low concentrations of fluoride in saliva and dental plaque fluid can facilitate remineralization by stabilizing calcium and phosphate ions near the tooth surface, thereby enhancing the redeposition of minerals into partially demineralized enamel lesions [4]. However, this beneficial effect is not universal and may be attenuated under specific clinical conditions. For instance, when the dental plaque biofilm becomes excessively thick, fluoride diffusion into subsurface enamel is hindered, leading to an altered remineralization pattern. Additionally, reduced salivary flow, limited buffering capacity, and variations in biofilm matrix composition can further restrict the cariostatic potential of fluoride [1,2,3]. These limitations highlight the importance of considering patient-specific oral environments when interpreting laboratory findings and applying them in clinical practice. Beyond its influence on enamel chemistry, fluoride also possesses significant antibacterial properties, particularly against *Streptococcus mutans*, one of the primary etiological agents of dental caries [5]. Fluoride disrupts bacterial metabolism by inhibiting the enzyme enolase within the glycolytic pathway, leading to decreased acid production and diminished pathogenicity of cariogenic biofilms [3]. Under acidic conditions, fluoride can also diffuse into bacterial cells in its undissociated form as hydrogen fluoride (HF). Once inside, HF dissociates, causing intracellular acidification and disruption of enzymatic activity, further compromising bacterial survival and metabolic function [6,7]. Collectively, these mechanisms significantly reduce the cariogenic potential of oral biofilms and contribute to the prevention of dental caries. Nevertheless, it is essential to acknowledge that excessive fluoride exposure, whether through drinking water, toothpaste ingestion, or other systemic sources, can lead to adverse outcomes such as dental fluorosis, skeletal fluorosis, and potential neurotoxic effects, particularly when intake surpasses recommended thresholds [8,9]. Current evidence on systemic risks remains heterogeneous and sometimes conflicting, underscoring the need for well-designed longitudinal studies that can clarify dose–response relationships and vulnerable population groups. Therefore, balancing fluoride’s well-established benefits in caries prevention with considerations of safety, individual susceptibility, and evolving public health policies remains a critical focus in both contemporary dental practice and future research.

## 2. Fluoride-Based Products and Clinical Applications

Over the past several decades, various fluoride-based products have been developed for both professional and personal use in the prevention and management of dental caries. The choice of a particular fluoride modality depends on multiple factors, including patient age, individual caries risk, esthetic considerations, and the level of fluoride exposure in the local environment. Understanding the specific characteristics, mechanisms, and clinical implications of these products is critical for tailoring effective preventive strategies.

### 2.1. Fluoride Toothpaste

Among all fluoride delivery vehicles, fluoridated toothpaste remains the most accessible and widely used method for caries prevention [5]. Standard formulations for adults typically contain fluoride concentrations ranging from 1000 to 1500 ppm, delivered through compounds such as sodium fluoride (NaF), stannous fluoride (SnF_2_), or sodium monofluorophosphate (MFP). These formulations effectively promote remineralization and inhibit the demineralization process, contributing significantly to the decline in caries prevalence over recent decades [10]. However, variations in study design and population characteristics make it difficult to attribute the decline solely to fluoride toothpaste, and other public health measures may also have contributed. For children under six years of age, lower fluoride concentrations between 500 and 1000 ppm are recommended to minimize the risk of dental fluorosis, particularly since young children may inadvertently swallow toothpaste during brushing. According to the latest protocol of the American Academy of Pediatric Dentistry, the use of fluoride toothpaste should begin with the eruption of the first primary tooth, with parents or caregivers supervising the amount applied to reduce ingestion risk [11,12]. For adults, restorative dentistry textbooks specify that toothpaste should contain at least 1000 ppm fluoride to ensure caries-preventive efficacy, while other guidelines recommend 1350–1500 ppm as the standard range for optimal protection [5,13,14]. In addition to conventional formulations, high-fluoride toothpastes containing up to 5000 ppm fluoride are available by prescription for adults at elevated caries risk, such as those with xerostomia, orthodontic appliances, or a history of rampant caries [13]. Clinical guidelines emphasize using only a pea-sized amount of toothpaste and supervising brushing in young children to reduce systemic fluoride exposure [11,12]. Nevertheless, adherence to these recommendations in real-world settings remains inconsistent, particularly in low-literacy or resource-limited communities, which may reduce the effectiveness of these preventive strategies. Despite these precautions, twice-daily brushing with fluoridated toothpaste remains a cornerstone of modern caries prevention across all age groups [13], highlighting the importance of proper patient education and compliance in achieving optimal outcomes [14]. Yet, evidence suggests that compliance is strongly influenced by socioeconomic status and health literacy, indicating that toothpaste efficacy in clinical trials may not always translate into community-wide effectiveness. Additional fluoride compounds such as amine fluoride (AmF) and SnF_2_ should also be acknowledged. Amine fluoride acts more rapidly due to its surfactant properties and strong enamel adhesion [15], whereas MFP requires enzymatic hydrolysis and thus releases fluoride more slowly [16]. Stannous fluoride not only provides fluoride ions but also exerts antimicrobial activity through tin, offering broader protective effects [17]. Importantly, synergistic benefits have been observed when AmF is combined with SnF_2_, resulting in enhanced antibacterial and anti-demineralization properties [18]. Still, comparative trials evaluating the long-term superiority of these combined formulations over conventional fluoride toothpastes remain limited. Accessibility to fluoridated toothpaste, however, differs substantially across regions worldwide. In most high-income countries, the majority of households have routine access to affordable fluoride toothpaste through retail markets and public health programs, which has contributed to significant reductions in caries prevalence. In contrast, surveys from low- and middle-income countries indicate that availability remains limited, with usage rates below 30% in some parts of sub-Saharan Africa and Southeast Asia due to cost, distribution challenges, and lack of regulatory standards. These disparities emphasize the need for global health initiatives to ensure equitable access to fluoride toothpaste as an essential preventive measure [19,20,21]. These global disparities underscore not only the importance of fluoride toothpaste access but also the need for culturally tailored health promotion programs to improve acceptance and sustained use. Future research may explore combining fluoride with additional remineralizing agents to enhance its preventive efficacy further. Fluoride mouthwashes serve as an adjunctive caries-preventive modality. Common over-the-counter formulations include 0.05% sodium fluoride (~230 ppm F) for daily use and 0.2% NaF (~900 ppm F) for weekly use in school-based programs. These rinses contain similar fluoride compounds to toothpaste, such as NaF, and are particularly beneficial for high-caries-risk patients by sustaining low-level fluoride exposure in the oral environment [22].

### 2.2. Fluoride Varnishes and Mouthrinses

Fluoride varnishes are professionally applied topical agents designed to deliver high concentrations of fluoride directly to the tooth surface. Typically formulated as 5% sodium fluoride varnish, they contain approximately 22,600 ppm fluoride. These varnishes adhere to enamel, allowing sustained fluoride release over several hours and enhancing remineralization while reducing cariogenic bacterial activity [23]. Nevertheless, reported effectiveness varies across studies, partly due to differences in application protocols, follow-up duration, and patient compliance, which complicates the direct comparison of outcomes. Clinical evidence demonstrates that fluoride varnish applications performed two to four times per year can reduce the incidence of dental caries in permanent teeth by up to 43% [5]. It should be noted, however, that this preventive effect is not uniform across populations, with some trials reporting more modest reductions, highlighting the need for standardized protocols and longer-term evaluation. Varnishes are particularly suitable for young children, including those under six years old, because they are professionally applied and minimize systemic ingestion. Despite this advantage, professional application increases costs and limits accessibility in low-resource settings, raising concerns about equity in preventive care. Additionally, varnishes offer significant benefits for patients with high caries risk, orthodontic appliances, or exposed root surfaces [4]. As fluoride varnish continues to gain recognition as a versatile tool in preventive dentistry, ongoing research is exploring the integration of calcium-phosphate additives to improve efficacy and patient acceptance. Yet, evidence regarding the clinical superiority of such modified varnishes remains preliminary, and further large-scale randomized trials are required before routine adoption. In contrast, fluoride mouthrinses are generally formulated at concentrations of 230 ppm for daily use or 900 ppm for weekly applications. They are effective in reducing caries incidence, particularly in individuals at high risk for dental caries. However, these rinses are generally not recommended for children younger than six years due to the increased risk of ingestion and potential systemic toxicity [3,12]. Moreover, patient adherence to regular rinsing regimens is often suboptimal, which may diminish real-world effectiveness despite strong evidence from controlled studies.

### 2.3. Fluoride Supplements (Tablets and Drops)

Fluoride supplements, available as tablets or drops, are intended for systemic use in regions lacking adequate community water fluoridation or where natural fluoride levels are insufficient. The prescribed dosage varies based on a child’s age and the fluoride concentration in the local water supply, usually ranging from 0.25 to 1.0 mg per day [12]. When taken during tooth development, systemic fluoride can integrate into hydroxyapatite, enhancing enamel resistance to acid dissolution [3]. Nonetheless, the actual cariostatic benefit of systemic supplementation has been inconsistent across studies, and much of the supporting evidence comes from older trials with methodological limitations. However, careful monitoring is essential to prevent excessive fluoride intake, which increases the risk of dental fluorosis. This concern has fueled ongoing debates regarding the ethical balance between population-level caries prevention and the potential for iatrogenic harm, particularly in young children. Consequently, the use of systemic fluoride supplements has declined in many regions, as topical fluoride modalities offer effective caries prevention with a lower risk of systemic exposure. Moreover, declining prescription rates in high-income countries contrast with continued use in some low- and middle-income regions, reflecting disparities in public health policies and resource availability. Despite their efficacy in specific high-risk populations, the preference has shifted toward topical approaches whenever feasible, underscoring the need for individualized assessments of total fluoride exposure. Future research should focus on refining guidelines for systemic supplementation, incorporating factors such as dietary fluoride intake, regional water fluoridation variability, and genetic susceptibility to fluorosis.

### 2.4. Silver Diamine Fluoride (SDF)

SDF is a topical agent used for arresting active carious lesions and offers a non-invasive alternative for caries management, particularly in populations with limited access to restorative care [24]. Clinical evidence from systematic reviews and randomized controlled trials supports its high efficacy compared with other topical fluoride treatments, with studies highlighting its significant role in community-based caries management and preventive programs [25,26,27]. However, the magnitude of benefit has varied between studies, and most long-term data are derived from pediatric populations, leaving uncertainty about the durability of the effect in adults. Despite these advantages, one significant limitation of SDF is the black staining that occurs on arrested lesions, which may pose esthetic concerns, especially for anterior teeth. This esthetic concern often limits parental acceptance, and cultural attitudes toward visible staining further influence treatment uptake across different regions. Nonetheless, SDF remains a transformative tool in minimally invasive dentistry, emphasizing the importance of comprehensive patient and parent counseling to manage expectations regarding esthetic outcomes. Ongoing research aims to develop formulations that preserve SDF’s caries-arresting potential while minimizing discoloration [25,26,27]. Yet, current experimental formulations have shown mixed results, and large-scale clinical validation is still lacking. In pediatric dentistry, SDF is strongly recommended, particularly for young or uncooperative children with early childhood caries, due to its proven efficacy and ease of application [25,26,27]. Nevertheless, questions remain about optimal reapplication intervals, cost-effectiveness compared with other preventive strategies, and integration into comprehensive caries management protocols. However, some restorative dentistry and caries textbooks for adults do not recommend routine SDF applications because of the esthetic drawback of black staining and the availability of restorative alternatives [24]. Nevertheless, SDF may be beneficial in adults with high caries risk, root caries, or those who are medically compromised and unable to receive conventional restorative treatments [24]. Future investigations should also explore patient-reported outcomes, long-term cost-effectiveness, and strategies to improve esthetic acceptability while retaining clinical efficacy.

### 2.5. Comparison of Fluoride Products

Numerous fluoride-based products are available for both professional and personal use, each differing in fluoride concentration, mechanism of action, clinical indications, and potential limitations [1,2,3]. Table 1 provides a comparative overview of these fluoride products, summarizing key aspects to assist clinicians in selecting the most appropriate intervention tailored to individual patient needs, caries risk, and practical considerations. However, head-to-head comparative trials remain scarce, and most data are derived from heterogeneous study designs, which limits the ability to establish clear superiority among products. While topical fluoride applications such as toothpaste and varnishes remain the primary preventive measures [13,25], fluoride gels (neutral or acidulated) also play a role in professional care, with variations in pH designed to balance enamel safety and fluoride uptake [28]. Yet, evidence regarding the clinical advantage of one formulation over another remains inconclusive, suggesting that practitioner preference often outweighs strong comparative data. Products such as MI Paste and MI Paste Plus (GC Tooth Mousse series) provide casein phosphopeptide–amorphous calcium phosphate (CPP-ACP) with or without fluoride, supporting remineralization and offering alternatives for patients with sensitivity or high caries risk [4,29,30,31]. Still, long-term outcomes and cost-effectiveness data for CPP-ACP-based products remain limited, and their role relative to conventional fluoride strategies is not fully established. Additionally, prophylaxis pastes applied after scaling procedures may contain fluoride and contribute to short-term enamel protection [32,33]. Newer modalities like silver diamine fluoride offer promising alternatives for caries arrest, particularly in populations with limited access to conventional restorative care [24,26,27]. However, esthetic concerns, especially staining associated with SDF, require careful consideration [27,34]. Acceptance also varies considerably across cultural and socioeconomic contexts, further complicating the integration of SDF into mainstream practice. Systemic fluoride supplements, while effective, have seen declining use due to the risk of fluorosis and the growing emphasis on topical interventions [9,10]. Nevertheless, debates persist regarding whether systemic supplementation should be re-evaluated in communities with poor access to topical products, highlighting the tension between efficacy, safety, and equity in global caries prevention. Nevertheless, they remain clinically relevant in areas lacking water fluoridation or where the dietary intake of fluoride is insufficient [35]. Ultimately, choosing the optimal fluoride strategy should be guided by evidence-based recommendations, patient preferences, and practical considerations such as cost-effectiveness, availability, and patient compliance. Future research should prioritize comparative effectiveness studies that include patient-reported outcomes, cultural acceptability, and real-world cost–benefit analyses to better inform clinical and public health recommendations.

### 2.6. Emerging Fluoride Technologies

Beyond traditional fluoride modalities, emerging technologies are being explored to enhance caries prevention and improve patient outcomes. One promising avenue involves the use of nano-hydroxyapatite (nHAp), a biomimetic material that closely resembles the natural mineral composition of tooth enamel. Studies suggest that nHAp can effectively occlude enamel porosities, promote remineralization of early carious lesions, and reduce dentin hypersensitivity [36,37]. However, much of the evidence is derived from short-term or in vitro studies, and the durability of these effects in long-term clinical use remains uncertain. When used alongside fluoride, nHAp may contribute to remineralization, although current evidence indicates that fluoride remains the primary agent for caries prevention [4]. This underscores that nHAp should be viewed as a potential adjunct rather than a replacement, pending stronger comparative clinical data. Another emerging approach involves fluoride-containing bioactive glass formulations. Fluoride-containing bioactive glasses have shown promise in in vitro studies for promoting enamel remineralization, although robust clinical data remain limited, and further research is needed to validate their efficacy compared with established fluoride therapies [3,4]. At present, the lack of randomized controlled trials limits their integration into evidence-based guidelines. Moreover, research has focused on innovative delivery systems, such as sustained-release varnishes and polymeric carriers designed to prolong fluoride availability at the tooth surface [4]. These novel delivery vehicles aim to enhance the substantivity of fluoride and reduce the frequency of applications, which could improve patient compliance and long-term preventive effectiveness. Yet, these delivery systems often involve higher costs and greater manufacturing complexity, which may hinder widespread adoption, particularly in low-resource settings. Other experimental additives, such as silver nanoparticles and zinc oxide nanoparticles, are also under investigation for their antibacterial and cariostatic potential, although robust clinical data remain limited. Additionally, potential cytotoxicity and long-term biocompatibility of nanoparticles remain underexplored, warranting caution before clinical translation. In addition to these novel approaches, restorative materials themselves can act as fluoride reservoirs. Glass ionomer cements (GICs) were among the first restorative materials with inherent fluoride release, providing long-term anticariogenic effects and reducing the risk of secondary caries [38]. Nevertheless, the fluoride release from restorative materials tends to decline over time, and the true magnitude of their anticariogenic benefit in vivo is still debated. Advances in adhesive technology have also led to the development of fluoride-containing bonding agents capable of sustained fluoride release within the hybrid layer, offering added protection against demineralization at restoration margins [39]. Furthermore, fluoride-releasing composite resins are now available, which, although releasing fluoride at lower levels than GICs, still contribute to localized caries prevention around restorations [40]. Further comparative studies are needed to determine whether these materials provide clinically meaningful advantages over conventional composites. While preliminary data for these emerging technologies appear promising, significant challenges remain. These include higher production costs, limited availability, and a lack of robust clinical evidence directly comparing these technologies to well-established fluoride therapies. Future research should prioritize large-scale, well-designed clinical trials to determine whether these newer modalities can achieve comparable or superior outcomes in caries prevention, particularly in high-risk populations. Nonetheless, emerging fluoride technologies hold substantial promise for improving both the efficacy and esthetics of caries prevention, potentially offering alternatives for patients who have concerns about the drawbacks of existing fluoride treatments, such as staining or taste alterations. Ultimately, without rigorous cost-effectiveness analyses and patient-reported outcome studies, the clinical role of these emerging technologies will remain uncertain.

## 3. Clinical Evidence and Efficacy of Fluoride Interventions

### 3.1. Clinical Outcomes of Topical Fluoride Interventions

Substantial clinical evidence confirms the caries-preventive effectiveness of topical fluoride measures. Meta-analyses indicate that toothpaste containing ≥1000 ppm fluoride reduces caries incidence by approximately 23% in children and adolescents compared with non-fluoridated toothpaste, with additional benefits observed at higher concentrations, particularly in high-risk populations [5,13]. However, heterogeneity across trials, including variations in outcome measures, study duration, and patient compliance, makes it difficult to establish precise dose–response relationships. A Cochrane review reported that professionally applied fluoride varnish can reduce caries incidence by 37% in primary dentition and by 43% in permanent dentition when applied two to four times annually [41]. Despite these promising results, questions remain about the optimal frequency and long-term sustainability of these effects in real-world clinical settings. Another Cochrane review demonstrated that fluoride mouthrinses reduce caries incidence by about 27% in the permanent teeth of children and adolescents, confirming their value as an adjunctive preventive strategy [22]. Yet, adherence to regular rinsing regimens is often suboptimal, especially among younger children, which may reduce effectiveness outside of controlled trial conditions. Furthermore, a systematic review and meta-analysis has shown that SDF is highly effective in arresting dentine caries in primary teeth, offering a valuable treatment option for pediatric populations [27]. Nevertheless, evidence in permanent dentition and adult populations is comparatively limited, and concerns about esthetic staining continue to affect broader acceptance. Taken together, evidence from multiple Cochrane reviews and systematic analyses consistently demonstrates that topical fluoride interventions, including toothpaste, varnishes, mouthrinses, and SDF, play a central role in effective caries prevention across diverse patient groups. Still, most available evidence comes from studies in children and adolescents, with fewer robust data for older adults, medically compromised patients, or populations in low-resource settings. The summarized efficacy of these fluoride interventions across different studies is presented in Table 2. Future research should therefore aim to address these gaps through multicenter trials that include diverse populations, standardized outcome measures, and patient-reported outcomes.

### 3.2. Comparative Efficacy: SDF vs. Other Interventions

Clinical trials and systematic reviews have established SDF as a highly effective agent for arresting active carious lesions, particularly in populations with limited access to restorative dental care. A systematic review by Gao et al. found that 38% SDF achieved caries arrest in approximately 81% of treated lesions in primary teeth, a notably higher efficacy than that typically observed with sodium fluoride varnishes under similar conditions [25]. Nevertheless, differences in study design, lesion activity assessment, and follow-up duration complicate direct comparisons between interventions. Randomized controlled trials have also demonstrated that SDF yields significantly higher caries-arrest rates than NaF varnish in moderate-to-deep dentinal lesions, although esthetic concerns related to black staining remain a limitation [26]. This drawback has been shown to significantly influence parental acceptance, with uptake varying across cultural and socioeconomic contexts. Furthermore, a meta-analysis by Chibinski et al. reported pooled caries-arrest rates of around 66% for SDF over follow-up periods of up to 12 months [27]. Longer-term evidence beyond two years, however, is limited, leaving uncertainty about the durability of caries arrest with repeated applications. These data emphasize that while both SDF and traditional fluoride varnishes are effective, SDF may offer superior efficacy in managing established carious lesions, particularly in high-risk populations. However, in community-based programs, the esthetic drawback of black staining may reduce parental acceptance and influence broader adoption, whereas in clinical settings SDF is often more readily accepted due to its proven efficacy and simplicity of application. Thus, the balance between clinical efficacy and esthetic acceptability remains a critical consideration in determining the broader adoption of SDF. Future studies should therefore include patient-reported outcomes and cost-effectiveness analyses to clarify contexts in which SDF provides the greatest benefits relative to traditional varnishes.

### 3.3. Efficacy in High-Risk Populations

Specific populations derive significant benefits from fluoride interventions. Older adults, for example, are particularly susceptible to root caries due to factors such as gingival recession and xerostomia. Evidence indicates that biannual applications of fluoride varnish or daily use of high-fluoride toothpaste can arrest or reverse the progression of root carious lesions [42]. Still, much of this evidence comes from relatively small cohorts, and the generalizability to diverse older populations with multiple comorbidities remains uncertain. In patients undergoing orthodontic treatment, fluoride interventions such as varnishes and high-fluoride toothpastes have been shown to effectively reduce the occurrence of white spot lesions associated with fixed appliances. Yet, compliance with fluoride regimens during orthodontic treatment is highly variable, which may diminish effectiveness outside of controlled trial conditions. Reviews have reported significant reductions in enamel demineralization when fluoride products are integrated into orthodontic care protocols [45]. Among individuals with special healthcare needs, including young children with early childhood caries (ECC) or patients who are unable to tolerate conventional restorative procedures, SDF serves as a valuable non-invasive alternative. Studies have demonstrated high rates of caries arrest with SDF, even in community-based settings [25]. Nevertheless, the absence of standardized protocols for reapplication and the lack of long-term follow-up data represent important research gaps.

### 3.4. Uncertainties and Future Research Directions

Despite extensive evidence supporting fluoride’s effectiveness in preventing dental caries, certain areas remain under investigation. Materials such as fluoride-containing bioactive glasses have demonstrated the ability to promote remineralization and reduce dentinal hypersensitivity in laboratory studies, but clinical data confirming clear advantages over conventional fluoride therapies are still limited [3]. Moreover, most available evidence is derived from in vitro or short-term trials, raising concerns about their translational value to routine dental practice. In addition, esthetic concerns associated with SDF, particularly the black staining of treated lesions, continue to pose challenges for broader acceptance, especially in anterior teeth. Acceptance is also influenced by cultural and socioeconomic factors, suggesting that esthetic concerns cannot be addressed solely through material modification but must also involve patient-centered counseling strategies. Research is ongoing to develop modified formulations or complementary treatments to mitigate these esthetic issues, although robust clinical trials are still needed to validate such approaches. At present, small pilot studies provide promising but inconclusive results, and there is a clear need for multicenter randomized controlled trials with standardized outcome measures. Future research should focus on long-term clinical studies to determine whether such emerging materials and technologies provide significant benefits beyond those achieved with established fluoride interventions. In particular, comparative effectiveness research is essential to clarify whether new technologies add meaningful clinical value or simply duplicate the effects of traditional fluoride modalities at higher costs. Improving patient compliance and addressing socioeconomic barriers also remain important goals to enhance the effectiveness of caries prevention strategies in diverse populations [3]. Addressing these socioeconomic barriers also requires policy-level interventions, including subsidy programs, public health education, and strategies to ensure equitable access to preventive care.

## 4. Target Populations, Acceptance Barriers, and Cultural Considerations

While fluoride’s preventive efficacy is well-established, real-world success depends heavily on whether patients accept these interventions and on how sociocultural factors influence perceptions and behaviors toward fluoride use. Understanding barriers and facilitators unique to different populations is essential for optimizing preventive strategies. In clinical practice and public health policy, it is clear that technical effectiveness alone does not guarantee widespread use; acceptance, education, and cultural adaptation are equally critical. Yet, systematic evaluations of how these sociocultural factors specifically alter fluoride uptake across different populations remain scarce, underscoring the need for more interdisciplinary research that combines dentistry, behavioral science, and public health.

### 4.1. Perceptions and Acceptance in Children and Parents

Parental concerns significantly influence fluoride use in children. Although fluoride varnishes and SDF offer effective caries prevention, esthetic issues, particularly the black staining associated with SDF, often create hesitation among parents, especially for visible anterior teeth. A UK study found that over 60% of parents initially rejected SDF treatment due to concerns about discoloration, but education about its benefits substantially increased acceptance rates [46]. Effective communication emphasizing the preventive benefits of fluoride while openly discussing potential cosmetic outcomes remains crucial for improving parental acceptance. Clinicians often find that when parents understand that discoloration signals the arrest of decay, they become more willing to accept treatment despite esthetic drawbacks. Still, most studies assessing parental acceptance have short follow-ups, and the long-term sustainability of such acceptance after repeated treatments is unclear. In practice, involving parents in shared decision-making and providing visual examples of treatment outcomes can be highly effective.

### 4.2. Older Adults: Underserved Needs and Health Literacy

Older adults frequently experience barriers to fluoride use that extend beyond the clinical effectiveness of fluoride products. For many, limited mobility, chronic diseases, cognitive impairment, and reduced manual dexterity decrease their ability to maintain oral hygiene or access professional preventive care. Cultural factors also play a role; in some populations, older adults may undervalue dental health relative to other medical conditions, perceiving tooth loss as an inevitable part of aging [47]. Addressing these perceptions through targeted education and simplified preventive regimens may enhance fluoride uptake in this group. Dental teams can collaborate with medical and community care providers to integrate oral health into overall health assessments, ensuring fluoride applications are included in routine care for older patients. Future research should also examine cost-effectiveness and caregiver burden, as these factors strongly influence the feasibility of preventive programs in geriatric populations.

### 4.3. Esthetic Concerns in Orthodontic and Adolescent Patients

Among adolescents, appearance and peer perceptions strongly influence willingness to accept treatments such as SDF or visible fluoride varnish applications. Studies indicate that esthetic concerns can outweigh the preventive benefits in this age group, leading some patients to decline fluoride treatments that might leave visible residue or staining [46]. Involving adolescents directly in treatment discussions and providing discreet options, such as clear varnishes, may help improve acceptance. Dental professionals also find that explaining the long-term benefits of fluoride in preventing permanent white spot lesions or enamel damage can persuade adolescents to prioritize health over temporary esthetic concerns. Nevertheless, behavioral interventions targeting this age group remain underexplored, and evidence on effective communication strategies tailored to adolescents is limited.

### 4.4. Special Healthcare Needs: Behavioral and Practical Challenges

For patients with special healthcare needs, barriers to fluoride treatment often relate to behavioral challenges rather than cultural attitudes. In this context, special healthcare needs refer to individuals with systemic or genetic disorders such as Down syndrome, cerebral palsy, or intellectual disabilities; those with physical or neuromotor limitations that hinder independent toothbrushing; and medically compromised or behaviorally challenged individuals, including patients with dementia or those requiring long-term institutional care. Short treatment times and minimal invasiveness make SDF and other fluoride treatments valuable tools for these groups. However, caregivers may still hesitate due to unfamiliarity with these newer modalities or concerns about esthetics and safety [46]. Training healthcare providers to counsel caregivers effectively is key to broader adoption. Practical strategies, such as using calm communication, visual support, and incremental treatment steps, can help improve cooperation and reduce stress for patients and caregivers alike. However, there is a paucity of large-scale studies evaluating fluoride interventions in these populations, leaving uncertainty about optimal protocols and long-term outcomes.

### 4.5. Cultural and Socioeconomic Differences

Acceptance of fluoride treatments varies significantly across cultures and socioeconomic contexts. In Western countries, cosmetic outcomes often play a central role in treatment decisions, whereas studies in several Asian populations suggest that parents may prioritize disease prevention over esthetic considerations [46]. Socioeconomic status also strongly influences the availability and use of fluoride-based preventive care. Evidence demonstrates that community water fluoridation not only reduces overall dental caries prevalence but also narrows oral health disparities among lower-income groups [43]. Systematic reviews further confirm that school-based fluoride mouthrinse programs can effectively lower caries incidence, particularly in resource-limited settings where access to other fluoride sources is restricted [22]. Importantly, strategies must be tailored to reflect cultural values and address barriers such as cost, accessibility, and health literacy in order to maximize acceptance and sustainability of preventive programs. Regional variations among developing countries highlight both the opportunities and challenges in implementing fluoride interventions. For example, the World Health Organization has supported salt fluoridation initiatives in Mexico, Costa Rica, and Colombia, where centralized water fluoridation is less feasible, and these programs have demonstrated substantial reductions in caries prevalence [20]. In contrast, experiences from African countries highlight more heterogeneous outcomes. While efforts to improve fluoride availability and oral health promotion exist, persistent barriers such as limited infrastructure, economic inequality, and low population awareness continue to hinder large-scale progress [19,21]. These examples underscore the importance of adapting fluoride delivery models to local cultural, social, and economic contexts rather than applying a uniform global approach. Comparative studies across countries with different delivery models are still limited, and more rigorous evaluations are needed to identify which culturally tailored interventions achieve the best balance between effectiveness, acceptability, and sustainability.

## 5. Clinical Recommendations and Implementation Strategies

### 5.1. Individualized Fluoride Recommendations

Effective prevention of dental caries requires tailoring fluoride strategies to each patient’s risk profile, age, and clinical condition. Current guidelines emphasize individualized care, recognizing that patients at higher caries risk, such as those with frequent sugar intake, orthodontic appliances, or xerostomia, benefit from more intensive fluoride interventions including professionally applied varnishes or high-fluoride toothpastes containing up to 5000 ppm fluoride [35,48,49]. However, evidence comparing the relative effectiveness of these intensive regimens remains limited, and most recommendations are extrapolated from short-term trials. In contrast, individuals at low risk may achieve sufficient protection with standard fluoride toothpaste alone. Recommended systemic intake also varies by age group. For infants and young children, a safe intake is approximately 0.05 to 0.07 mg F/kg body weight per day, with supervised use of low-fluoride toothpaste (500–1000 ppm) to reduce the risk of fluorosis [12,35]. The balance between maximizing preventive benefits and minimizing fluorosis risk continues to generate debate, particularly in regions without water fluoridation. For school-aged children and adolescents, toothpaste containing 1000–1500 ppm fluoride is appropriate, whereas high-fluoride products (up to 5000 ppm) are reserved for those with elevated caries risk [13,42]. In adults, adequate dietary fluoride intake is set at about 3 mg/day for women and 4 mg/day for men, with a tolerable upper intake level of 10 mg/day established by the Institute of Medicine [50]. Yet, population-level monitoring of actual fluoride intake is rarely performed, and potential cumulative exposure from diet, drinking water, and dental products remains insufficiently quantified. In patients with systemic conditions such as diabetes, who are at increased risk of both caries and periodontal disease, regular topical fluoride applications are recommended as an essential preventive measure. Twice-daily brushing with fluoridated toothpaste, supplemented by professionally applied fluoride varnish or gel when indicated, can effectively enhance enamel resistance and reduce caries susceptibility. Nevertheless, patient adherence to these intensified regimens is variable, and long-term outcomes under real-world conditions are less well documented. The frequency and intensity of topical fluoride use should be tailored to individual caries activity and overall oral health status [51,52]. In pregnant women, the routine use of topical fluoride, such as brushing with fluoride toothpaste or applying fluoride varnish, is considered safe and effective for maintaining oral health. However, systemic fluoride supplementation, including tablets or drops, is generally not recommended because of insufficient evidence of preventive benefit and potential risks. Preventive strategies should instead emphasize maintaining optimal oral hygiene with standard fluoride toothpaste while avoiding unnecessary systemic fluoride exposure during pregnancy [53,54]. Further research is also needed to evaluate subtle developmental or systemic effects, as current recommendations are largely based on precautionary principles rather than robust clinical data. This patient-centered approach aligns with the principles of minimally invasive dentistry, prioritizing disease control over restorative interventions [55]. Furthermore, clinicians are encouraged to incorporate patient preferences, values, and social circumstances into preventive strategies, as shared decision-making fosters trust and improves adherence to long-term recommendations [55,56]. Still, practical tools to implement shared decision-making in busy clinical environments remain underdeveloped, representing an area for future innovation. To provide a clearer overview for clinicians, we have summarized individualized recommendations for different at-risk populations and their corresponding fluoride modalities in Table 3.

### 5.2. Protocols for Clinical Practice

Professional application of fluoride varnish two to four times annually has been shown to significantly reduce caries incidence, with Cochrane reviews indicating reductions of up to 37% in permanent dentition and 43% in primary dentition when applied regularly in children and adolescents [41]. Yet, variations in application intervals, operator technique, and follow-up duration across trials suggest that the magnitude of benefits may differ in real-world practice. For home care, daily use of toothpaste containing at least 1000 ppm fluoride remains the standard recommendation, while higher concentrations should be reserved for patients with specific clinical indications. Importantly, in pediatric care, supervision is crucial to prevent excessive ingestion and reduce the risk of dental fluorosis, reinforcing guidelines to use only a pea-sized amount of toothpaste [49,60]. Nevertheless, adherence to these recommendations outside controlled environments is inconsistent, and more pragmatic studies are needed to evaluate their impact on fluorosis prevalence. In addition, scheduling follow-up visits to evaluate patient compliance and reinforce preventive advice is important for sustaining the benefits of fluoride treatments over time. Clinicians should avoid assuming that patients will consistently use recommended products without regular encouragement and clear, practical instructions. Without sustained behavioral reinforcement, even evidence-based protocols risk losing effectiveness, underscoring the need for ongoing patient engagement strategies.

### 5.3. Implementation in Public Health and Overcoming Barriers

Translating clinical evidence into practice requires integrating fluoride interventions into broader public health initiatives. Community water fluoridation has been recognized as a landmark public health achievement, contributing to substantial reductions in dental caries and narrowing disparities in oral health, particularly among socioeconomically disadvantaged populations [43]. Similarly, school-based fluoride programs, including mouthrinse [22] or varnish applications [41], have demonstrated an efficacy in reducing caries increments, especially in populations lacking access to routine dental care. However, the scalability and sustainability of such programs remain uncertain, particularly in low-resource settings where workforce shortages and inconsistent funding are common. However, significant barriers remain, including esthetic concerns related to SDF, which can affect patient acceptance, particularly in Western populations where cosmetic appearance often influences health decisions [46]. This indicates that esthetic concerns are not merely individual preferences but reflect deeper cultural values that must be addressed in program design. Beyond esthetic issues, practical barriers such as cost, access, and health literacy can hinder widespread adoption of fluoride interventions. Another practical consideration is ensuring the affordability of fluoride products, especially high-fluoride toothpastes and professional varnish applications, which can be prohibitively expensive for some patients. Public health policies and reimbursement structures should strive to reduce these financial barriers to ensure equitable access to preventive care [48]. Still, there is limited economic evaluation of fluoride interventions at the population level, and cost-effectiveness data are essential to guide reimbursement policies. Communication skills are equally critical in overcoming these challenges. Using clear, simple language, avoiding technical jargon, and providing visual aids can significantly enhance patient understanding, particularly in diverse communities with varying literacy levels [56,61]. Ultimately, successful public health strategies must blend scientific evidence with practical, culturally sensitive approaches tailored to local community contexts.

### 5.4. Future Perspectives

Emerging fluoride technologies, including sustained-release varnishes, fluoride-containing bioactive glasses, and biomimetic materials such as nano-hydroxyapatite, show promise in enhancing enamel and dentin remineralization and potentially reducing the frequency of professional applications [36,62]. However, the current body of clinical evidence supporting these innovations remains limited, and further high-quality trials are required before these materials can be confidently integrated into routine practice [55]. The lack of standardized outcome measures across emerging fluoride technologies also hampers meaningful comparison with conventional interventions. While innovation is crucial for advancing preventive dentistry, clinicians should critically evaluate new products and avoid adopting unproven alternatives prematurely. Remaining cautious ensures patient safety and maintains trust in professional recommendations. At the same time, innovation should be accompanied by transparent reporting of conflicts of interest, as many emerging products are industry-driven. Ultimately, preventive strategies are most effective when seamlessly integrated into routine clinical workflows and presented as an essential component of comprehensive oral healthcare, rather than as optional or standalone interventions. Future work should focus not only on novel materials but also on optimizing delivery systems and integrating preventive strategies within broader oral health promotion frameworks.

## 6. Health Risks, Controversies, and Public Health Perspectives

### 6.1. Health Risks Associated with Fluoride Exposure

Despite fluoride’s proven efficacy in caries prevention, concerns persist regarding potential adverse health effects arising from excessive intake. Dental fluorosis is the most commonly observed consequence, resulting from excessive fluoride ingestion during enamel formation. It manifests as subsurface hypomineralization, producing white opacities, mottling, and, in severe cases, brown staining and enamel pitting. Data from the U.S. National Health and Nutrition Examination Survey (NHANES) 1999–2004 indicated that approximately 23% of adolescents exhibited some degree of dental fluorosis, with about 2% showing moderate fluorosis and fewer than 1% showing severe forms, underscoring the cumulative exposure from multiple fluoride sources such as drinking water, toothpaste ingestion, and dietary supplements [63]. Importantly, the clinical significance of mild fluorosis remains debated, with some viewing it as primarily cosmetic while others note possible psychosocial consequences. Skeletal fluorosis is another documented health concern, primarily observed in regions where drinking-water fluoride levels substantially exceed recommended standards. Chronic ingestion of fluoride at concentrations typically above 3–6 mg/L can result in increased bone density, joint pain, stiffness, and, in advanced stages, skeletal deformities and disability. Endemic skeletal fluorosis remains a significant public health issue in regions such as India and China, where high fluoride levels in drinking water have led to widespread cases [64]. However, the condition is exceedingly rare in areas where drinking-water fluoride concentrations remain below 1.5 mg/L, the guideline established by the World Health Organization [64]. Emerging research has also investigated potential neurodevelopmental impacts of fluoride exposure. A widely cited meta-analysis by Choi et al. analyzed 27 epidemiological studies and reported an association between higher fluoride exposure and lower intelligence quotient (IQ) scores among children living in areas with naturally high fluoride concentrations [8]. A longitudinal cohort study in Mexico found that higher prenatal fluoride exposure was associated with lower cognitive outcomes in children aged 4 to 12 years [65]. However, most of these studies relied on ecological designs with limited control for confounders, making it difficult to infer causality at fluoridation levels used in public health programs. It is noteworthy that most studies suggesting neurotoxicity were conducted in regions with fluoride levels substantially exceeding those used in regulated water fluoridation programs. Potential effects of fluoride on endocrine function, including thyroid health, have been explored in the scientific literature. Some research has suggested associations between elevated fluoride intake and alterations in thyroid hormone levels, particularly in populations with insufficient iodine intake. However, evidence remains limited and inconclusive, and further research is needed to determine whether fluoride exposure at levels used in public health interventions has significant endocrine impacts [9].

### 6.2. Controversies and Ethical Considerations

Fluoride’s public health application, especially through community water fluoridation (CWF), has generated ethical debates and public opposition in certain regions. Proponents of CWF emphasize its substantial benefits in reducing caries prevalence and improving oral health equity, particularly in lower-income populations who may lack access to dental care or fluoride-containing products [43]. Conversely, critics raise concerns regarding individual autonomy, arguing that mass-fluoridation programs impose treatment without explicit consent, thereby conflicting with the ethical principle of informed choice [66]. This reflects the broader ethical tension in preventive medicine between maximizing collective benefits and preserving individual autonomy. Numerous cost–benefit analyses support the economic advantages of CWF, noting significant savings in dental treatment costs attributable to reduced caries incidence [67]. The Nuffield Council on Bioethics concluded that decisions regarding water fluoridation should be determined through local democratic processes, balancing public health benefits against individual rights and preferences [68]. In addition to water fluoridation, salt fluoridation has been introduced as an alternative mass-fluoridation strategy in countries such as Switzerland, Mexico, and Costa Rica. While widely regarded as effective and cost-efficient, salt fluoridation raises similar ethical debates concerning informed consent, population-wide exposure, and the challenge of tailoring fluoride intake to individual needs. Proponents argue that salt fluoridation is particularly valuable in countries with fragmented water supply systems, whereas critics note that it may disproportionately affect individuals with dietary restrictions or those at higher risk of overexposure [20,44]. The scientific debate is further complicated by methodological challenges in fluoride research. Critics argue that earlier studies supporting community fluoridation may suffer from confounding factors, while studies indicating harm are often criticized for poor study design, inadequate exposure assessment, and failure to account for confounders such as socioeconomic status or co-exposures [9]. This polarization complicates policymaking, as governments must act despite incomplete evidence while also addressing strong public skepticism amplified through advocacy and media. This scientific polarization contributes to persistent public skepticism and hinders clear communication regarding fluoride’s risks and benefits.

### 6.3. Global Public Health Perspectives

Global perspectives on fluoride utilization exhibit considerable variation due to differences in cultural attitudes, regulatory frameworks, and local geological conditions. In the United States, a substantial proportion of the population receives fluoridated water, with the U.S. Public Health Service recommending a fluoride concentration of 0.7 mg/L to achieve an optimal balance between caries prevention and the risk of dental fluorosis [43,69]. In contrast, numerous European countries have opted against widespread implementation of CWF, instead favoring targeted interventions such as salt fluoridation, milk fluoridation, or topical fluoride applications. Countries including Germany, Sweden, and Switzerland have discontinued water fluoridation programs, citing ethical considerations, low prevalence of dental caries, and public opposition [20]. Case studies provide critical insights into the public health implications of policy shifts regarding fluoridation. For example, the discontinuation of CWF in Calgary, Canada, in 2011 was followed by an observed increase in the prevalence of dental caries among children [70]. This increase, combined with substantial debate and new epidemiological findings, ultimately prompted the reinstatement of fluoridation in 2021. This case illustrates how policy reversals create natural experiments but also highlights the difficulty of disentangling fluoridation effects from broader social and behavioral determinants of oral health. Conversely, in regions affected by endemic fluorosis, national programs prioritize defluoridation technologies and alternative fluoride delivery methods to mitigate excessive exposure while preserving the preventive benefits against dental caries [64,71]. Contemporary policy challenges encompass the accurate assessment of cumulative fluoride exposure from diverse sources and the necessity for individualized risk evaluation, particularly in vulnerable populations. Advances in biomarker research, including the analysis of fluoride concentrations in biological matrices such as nails and urine, offer potential for more precise exposure assessment and could inform personalized public health recommendations [72]. Yet, standardization of biomarker use and validation across diverse populations remain major challenges before they can guide policy. Furthermore, there is growing scientific interest in alternative caries-preventive strategies, such as the use of probiotics. However, current evidence for these approaches remains limited compared with the robust body of evidence supporting the established role of fluoride in public health interventions [73]. In summary, while fluoride continues to be recognized as an essential element in the prevention of dental caries, the ongoing evaluation of its benefits relative to potential risks remains a subject of scientific discourse and public deliberation. Future research and policy initiatives must endeavor to refine exposure guidelines, ensure transparent and effective communication, and respect community values to optimize the public health benefits of fluoride while safeguarding individual health.

## 7. Conclusions

Fluoride remains a cornerstone of modern dentistry for preventing and managing dental caries, supported by robust scientific evidence demonstrating its ability to enhance enamel remineralization, inhibit demineralization, and suppress cariogenic bacteria. A wide range of fluoride-based products, including toothpastes, varnishes, mouthrinses, supplements, and silver diamine fluoride, have proven effective across different populations and risk groups. Silver diamine fluoride, in particular, shows a high efficacy for arresting active carious lesions and is especially valuable for individuals with limited access to restorative care, despite concerns regarding staining. Acceptance of SDF also varies across cultural and socioeconomic contexts, which limits its universal applicability and requires further evaluation in diverse populations. At the same time, the use of fluoride requires careful attention to balance benefits with potential risks, such as dental and skeletal fluorosis or possible neurodevelopmental effects at excessive systemic exposure. Yet, most evidence on systemic risks comes from regions with naturally high fluoride levels, and data specific to controlled fluoridation programs remain sparse, underscoring the need for prospective longitudinal studies. Cultural perceptions, esthetic considerations, and ethical debates further influence public acceptance and highlight the importance of individualized care and effective communication.

Emerging technologies, such as nano-hydroxyapatite, fluoride-containing bioactive glasses, and advanced delivery systems, hold promise for improving remineralization, reducing application frequency, and enhancing patient compliance. Nevertheless, their higher production costs and limited availability currently restrict widespread use, raising questions about cost-effectiveness in real-world practice. However, current evidence for these newer options remains limited, and further rigorous clinical research is needed to establish their safety and efficacy compared with established fluoride therapies. Future research should refine safe exposure thresholds, optimize delivery methods, and investigate synergistic effects with other remineralizing agents. In addition, standardized outcome measures and patient-reported endpoints are needed to allow meaningful comparisons across different interventions. Equally important is addressing socioeconomic barriers and ensuring equitable access to fluoride-based care. The continued success of caries prevention globally will depend on integrating evidence-based guidelines with culturally sensitive public health strategies and innovative delivery models that enhance both acceptance and sustainability. Ultimately, future success will require bridging the persistent gap between controlled clinical trial efficacy and real-world effectiveness in diverse, resource-constrained settings.

## Figures and Tables

**Table 1:** Comparative overview of fluoride-based products.

Product Type	Fluoride Concentration	Mechanism of Action	Primary Indications	Limitations	Cost/Accessibility	References
Toothpaste	500–1500 ppm (standard); up to 5000 ppm in prescription formulations	Topical remineralization, antibacterial effects	Daily home use; high-fluoride pastes for adults at elevated caries risk	Risk of ingestion in young children; esthetic concern not relevant	Affordable and widely available in high-income settings, but significantly less affordable in low-income countries	[13,19,25]
Fluoride varnish	~22,600 ppm fluoride ions	Sustained topical release, promotes remineralization	Professional application for high-risk individuals	Potential taste issues, cost	Moderate to high cost; requires professional delivery; insurance coverage (e.g., Medicaid/ACA coverage) influences accessibility in certain settings	[13,25,32]
Fluoride gels (neutral NaF or acidulated APF)	Neutral: ~9000 ppm; Acidulated: ~12,300 ppm	High-concentration topical application; pH variations balance enamel safety and fluoride uptake	Professional use in patients with high caries risk	Risk of tissue irritation or fluorosis if misused	Moderate cost; generally accessible only in clinical environments; not for routine home use	[18,28]
Mouthrinse	230–900 ppm fluoride ions	Topical remineralization, antibacterial effects	High-caries-risk individuals	Not recommended for children under 6	Low to moderate cost; widely available in many regions, but school/community programs are limited in low-resource areas	[13,20,22,25]
Supplements (tablets/drops)	0.25–1.0 mg/day	Systemic incorporation during tooth development	Areas lacking fluoridated water supply	Risk of fluorosis if overdosed	Low cost, but availability dependent on public health policy; usage has declined in many regions	[13,25,34]
SDF	38% solution (approx. 44,800 ppm fluoride ions)	Arrests caries; antibacterial; promotes remineralization	Non-invasive caries management	Black staining of treated lesions	Relatively inexpensive; increasingly available in public health programs, but uptake limited by esthetic acceptance	[24,27]
MI Paste (GC Tooth Mousse)	No fluoride (CPP-ACP only)	Casein phosphopeptide–amorphous calcium phosphate supports remineralization	Patients with sensitivity, white spot lesions	Lacks fluoride; limited efficacy in high-risk patients	High cost; mainly available through private practices; limited public health access	[29,30]
MI Paste Plus (GC Tooth Mousse Plus)	900 ppm fluoride + CPP-ACP	Combines CPP-ACP with fluoride for enhanced remineralization	High caries-risk or sensitivity-prone patients	Not suitable for patients with milk protein allergy	Higher cost; limited availability outside private clinics; often not covered by public systems	[29,30]
Prophylaxis pastes (with fluoride)	1000–4000 ppm fluoride ions	Provides fluoride release after scaling, short-term enamel protection	Professional use following scaling/polishing	Temporary effect; not a long-term preventive measure	Moderate cost; only accessible via professional dental services	[35]

**Table 2:** Reported efficacy of fluoride interventions in preventing or arresting dental caries.

Fluoride Intervention	Concentration/Formulation	Reported Efficacy (Caries Reduction or Arrest)	Population Studied	References
Fluoride toothpaste	≥1000 ppm F	~23% reduction in caries incidence compared with non-fluoride toothpaste	Children and adolescents	[5,13]
High-fluoride toothpaste	5000 ppm F	Effective in arresting/reversing root caries; superior to standard toothpaste	Older adults and high-risk patients	[42]
Fluoride varnish	5% NaF (22,600 ppm)	37% reduction in primary dentition; 43% in permanent dentition (2–4 applications/year)	Children and adolescents	[41]
Fluoride mouthrinse	0.05% NaF daily (230 ppm) or 0.2% weekly (900 ppm)	~27% reduction in permanent teeth	School-aged children and adolescents	[22]
SDF	38% solution (~44,800 ppm F)	66–81% caries arrest in primary teeth	Children with ECC	[25,26,27]
Community water fluoridation	0.7 mg/L (USPHS guideline)	~25–30% reduction in caries prevalence	General population	[43]
Salt fluoridation	200–250 mg/kg	Substantial caries reduction; outcomes vary by country	School children in Mexico, Costa Rica, and Colombia	[44]

**Table 3:** Recommendations for fluoride use in different at-risk populations.

At-Risk Population	Recommended Fluoride Modality	Concentration/Frequency	Clinical Notes	References
Children (<6 years)	Fluoride toothpaste with parental supervision; fluoride varnish (professional)	500–1000 ppm F toothpaste twice daily; varnish 2–4 times/year	Minimizes fluorosis risk; avoid mouthrinse use	[11,12,41]
Adolescents with orthodontic appliances	High-fluoride toothpaste; fluoride varnish	1350–1500 ppm F daily; varnish every 3–6 months	Reduces enamel demineralization and white spot lesions	[42,45]
Older adults (xerostomia, root caries)	High-fluoride toothpaste; fluoride varnish	5000 ppm F toothpaste twice daily; varnish 2–4 times/year	Effective for arresting and reversing root caries	[42]
Pregnant women	Standard fluoride toothpaste; fluoride varnish if indicated	1000–1500 ppm F toothpaste twice daily	Systemic supplements (tablets/drops) not recommended	[53,54]
Patients with systemic diseases (e.g., diabetes)	Standard or high-fluoride toothpaste; professional varnish or gel	≥1500 ppm F toothpaste daily; varnish 2–4 times/year	Helps reduce caries risk; supports periodontal health	[51,52]
Oncology patients (head/neck radiotherapy)	High-fluoride gel or varnish; custom tray application	Daily gel trays (e.g., 5000 ppm) or varnish 3–4 times/year	Prevents radiation caries; domiciliary self-applied fluoride with tray shows effectiveness of up to ~70% in systematic review	[57,58,59]
Special healthcare needs (children with ECC, cognitive/physical impairment, elderly with dementia)	SDF; fluoride varnish	SDF annually or biannually; varnish adjunctively	Non-invasive, short application time; useful when compliance is limited	[25,26,27,46]

## Data Availability

No new data were created or analyzed in this study.
